# Cutaneous Microvascular Blood Flow and Reactivity in Hypoxia

**DOI:** 10.3389/fphys.2018.00160

**Published:** 2018-03-06

**Authors:** Benedikt Treml, Axel Kleinsasser, Karl-Heinz Stadlbauer, Iris Steiner, Werner Pajk, Michael Pilch, Martin Burtscher, Hans Knotzer

**Affiliations:** ^1^Department of General and Surgical Intensive Care, Medical University Innsbruck, Innsbruck, Austria; ^2^Department of Anesthesiology and Critical Care Medicine, Medical University Innsbruck, Innsbruck, Austria; ^3^Department of Anesthesiology and Critical Care Medicine, Klinikum Vöcklabruck, Vöcklabruck, Austria; ^4^Department of Pediatrics, County Hospital Kufstein, Kufstein, Austria; ^5^Department of Sport Science, Medical Section, University Innsbruck, Innsbruck, Austria; ^6^Department of Anesthesiology and Critical Care Medicine II, Klinikum Wels-Grieskirchen, Wels, Austria

**Keywords:** reactive hyperemia, flow motion, laser doppler flowmeter, hypoxia, cutaneous microvascular blood flow

## Abstract

As is known, hypoxia leads to an increase in microcirculatory blood flow of the skin in healthy volunteers. In this pilot study, we investigated microcirculatory blood flow and reactive hyperemia of the skin in healthy subjects in normobaric hypoxia. Furthermore, we examined differences in microcirculation between hypoxic subjects with and without short-term acclimatization, whether or not skin microvasculature can acclimatize. Fourty-six healthy persons were randomly allocated to either short-term acclimatization using intermittent hypoxia for 1 h over 7 days at an FiO_2_ 0.126 (treatment, *n* = 23) or sham short-term acclimatization for 1 h over 7 days at an FiO_2_ 0.209 (control, *n* = 23). Measurements were taken in normoxia and at 360 and 720 min during hypoxia (FiO_2_ 0.126). Microcirculatory cutaneous blood flow was assessed with a laser Doppler flowmeter on the forearm. Reactive hyperemia was induced by an ischemic stimulus. Measurements included furthermore hemodynamics, blood gas analyses and blood lactate. Microcirculatory blood flow increased progressively during hypoxia (12.3 ± 7.1–19.0 ± 8.1 perfusion units; *p* = 0.0002) in all subjects. The magnitude of the reactive hyperemia was diminished during hypoxia (58.2 ± 14.5–40.3 ± 27.4 perfusion units; *p* = 0.0003). Short-term acclimatization had no effect on microcirculatory blood flow. When testing for a hyperemic response of the skin's microcirculation we found a diminished signal in hypoxia, indicative for a compromised auto-regulative circulatory capacity. Furthermore, hypoxic short-term acclimatization did not affect cutaneous microcirculatory blood flow. Seemingly, circulation of the skin was unable to acclimatize using a week-long short-term acclimatization protocol. A potential limitation of our study may be the 7 days between acclimatization and the experimental test run. However, there is evidence that the hypoxic ventilatory response, an indicator of acclimatization, is increased for 1 week after short-term acclimatization. Then again, 1 week is what one needs to get from home to a location at significant altitude.

## Introduction

The issue how microcirculation and microcirculatory blood flow are influenced by a hypoxic stimulus is still a matter of debate and under investigation. One might assume—as also written in the scientific literature—that in hypoxia a decrease in oxygen delivery to the cells resulted in a vasodilation of the capillaries in order to enhance blood flow in the microcirculation (Martin et al., 1932; Granger et al., 1983; Delashaw and Duling, 1988; Davis et al., 2008). From animal experiments we know that microcirculatory blood flow in the skeletal muscle decreases during normobaric hypoxia and also that functional capillary density is significantly reduced (Saldívar et al., 2003). These observations were supported by other animal experiments reporting similar results (Fisher et al., 1992). Recently, nitric oxide levels were shown to increase during post-ischemic reperfusion in hamsters after 3 weeks of intermittent hypoxia (Bertuglia, 2008).

Data in humans are sparse. Recently, a small study in nine males showed hypoxia increasing epidermal hypoxia inducible factor-1α (HIF-1α) followed by nitrous oxide-mediated vasodilatation in skin. However, cutaneous hypoxia alone does not affect microcirculation (Siebenmann et al., 2017). Another small study in 11 subjects observed an increase in forearm skin blood flow after systemic hypoxia, without impact of local regulatory factors (Paparde et al., 2015).

Furthermore, a small study exposing four mountaineers to an altitude of 6,400 m examined their microcirculatory blood flow under the tongue semi-quantitatively (Martin et al., 2009). In these hypoxic subjects the mucosal microcirculatory blood flow was reduced, too. In contrast, Sherpas have recently been shown to have a higher sublingual microcirculatory blood flow and an increased capillary density at high altitude as compared to lowlanders (Gilbert-Kawai et al., 2017).

This paucity in knowledge on how microcirculation adapts to hypoxia and the question whether acclimatization may alter vascular reactivity led to conducting this investigation. We looked at reactive hyperemia in hypoxia and at the effect of hypoxic acclimatization.

Reactive hyperemia is the regular response of the microcirculation to an ischemic stimulus. The magnitude of this response mirrors the autoregulatory capacity of the microcirculation to counteract a decreased oxygen delivery. Until now, little is known how the microcirculatory reacts under already hypoxic conditions to ischemia to compensate oxygen debts.

This knowledge may help to understand pathomechanisms of ischemia/reperfusion injuries at high altitude.

In a second leg of this investigation we looked at the differences between those with hypoxic acclimatization compared to those without hypoxic preparation. An educated guess was that in hypoxia after hypoxic preparation there is a better adaptation of microcirculatory blood flow than without previous hypoxic exposure.

We thus hypothesized to

A.) detect the expected increase in microcirculatory blood flow in young healthy subjects under normobaric, hypoxic conditions. This also served to document a correct setup of this investigation.

B.) Furthermore, we sought to identify an improvement in the autoregulatory capacity of the microcirculation after an ischemic stimulus in an already hypoxic surrounding (null hypothesis),

C.) In a third step we sought to demonstrate that there is a difference in microcirculatory reactivity between those with and those without hypoxic acclimatization.

In summary, we sought to evaluate the ability of the skin microvasculature to acclimatize to hypoxia.

## Materials and methods

The ethical committee of the Innsbruck Medical University approved the study protocol (Protocol Number UN 4522/306/4.11). The study was performed as an amendment of a study investigating effects of a stay in a normobaric hypoxic chamber for 12 h between subjects with short-term short-term acclimatization in a normobaric, hypoxic chamber and control subjects (Niedermeier et al., 2017a,b; these two studies were embedded in a larger study already published, Burtscher et al., 2014).

### Study patients

**Figure 1:**
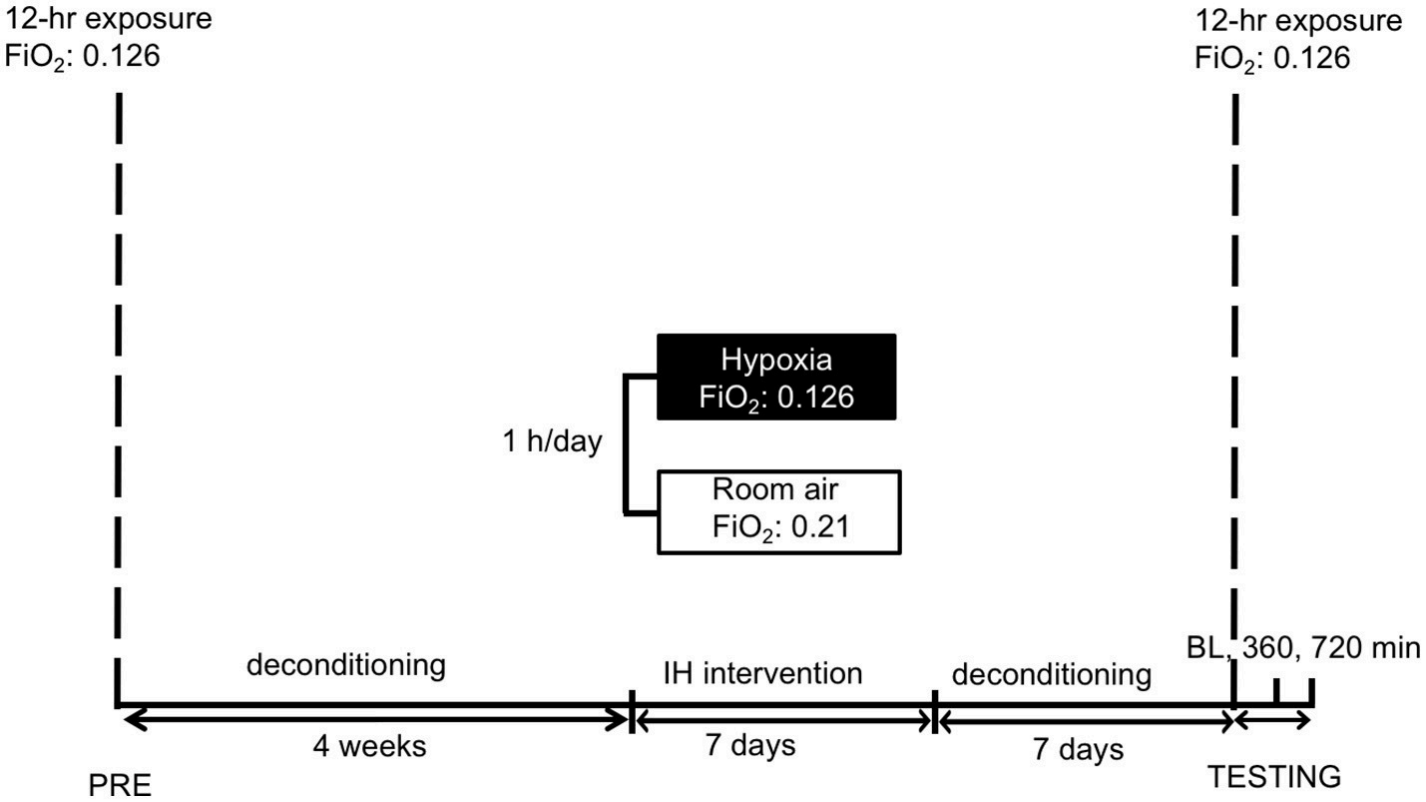
Study schedule: subjects were familiarized to hypoxia (12 h) followed by a 4-week period of deconditioning. Thereafter short-term acclimatization (1 h for 7 days) or sham-exposure were performed. After 1 week deconditioning the hypoxic exposure (12 h) with measurements followed. IH reflects intermittent hypoxia. FiO2 reflects fraction of inspired oxygen. BL, 360, 720 min reflect the time points of measurements, i.e., baseline before entering the hypoxic chamber and after 360 and 720 min in hypoxia, respectively.

Forty-six healthy female and male subjects were included in the study after a medical check. The participants were subjected to normobaric hypoxia (FiO_2_ = 0.126 equivalent to an altitude of 4.500 meters when applied in Innsbruck, 600 m) over 12 h (no physical activity) to familiarize with hypoxia and measurement procedures. After a de-acclimatization period of 4 weeks, subjects were allocated randomly into two groups (Figure 1). Unpublished observation from our group showed that a “wash out-period” of 1 month following a short exposure to hypoxia seems to be long enough to normalize the hypoxic ventilatory response (HVR) increase to baseline.

### Treatment group, controls, and protocols

One group was subjected to short-term acclimatization using intermittent passive hypoxic exposure at FiO_2_ = 0.126 over 1 h for 7 days (*n* = 23). We chose the 7 days-period between short-term acclimatization and hypoxic testing due to two main reasons. First (a) as in recent work of our group the HVR–seemingly the pivotal mechanism preventing acute mountain sickness-has been shown to be increased after 2 days of normoxia using a similar protocol of short term acclimatization (Wille et al., 2012). Furthermore, this increase in the HVR has been shown to last for up to 1 week, increasing SaO_2_ values up to 7% (Katayama et al., 2001, 2009). Second (b) 7 days are usually needed for the journey from home to base camp of a climbing destination.

Controls only received sham-acclimatization over 1 h for 7 days at an FiO_2_ of 0.209 (*n* = 23). During short-term acclimatization the participants could not perceive any group assignments (e.g., FiO_2_ values), furthermore a separate researcher not involved in the further analyses (M.P.) observed this segment.

After short-term acclimatization and sham-acclimatization respectively, 1 week of deconditioning preceded the actual experimental run. At “test day” all baseline measurements were obtained before entering the hypoxic chamber. There after all participants were subjected to normobaric hypoxic chamber for 12 h (i.e., 720 min). Measurements were obtained after 360 and 720 min in hypoxia, respectively.

Exclusion criteria were any overnight stays at altitudes >2.500 meters in the last 2 months. Any history of lung, cardiac, neurological, and psychiatric disease, respectively would have led to exclusion. For instance, chronic headache or history of migraine could have interfered with the results of the Lake Louise symptom scoring system (LLS). All subjects were told not to consume coffee or alcohol, any other drug and to refrain from exercising in the 24 h prior to the investigation. Written informed consent was obtained from all subjects.

### Experimental design

This study was designed as a double blinded randomized controlled trial.

Baseline measurements (BL) were performed at an altitude of 600 meters right before entering the hypoxic chamber. Microcirculatory blood flow and reactive hyperemia were investigated for obtaining individual baseline levels. Demographic data, Lake Louise Score (LLS) (Roach et al., 1993), arterial blood gas analysis, systemic arterial blood pressure and heart rate and arterial lactate values were also recorded.

The oxygen concentration in the normobaric hypoxia chamber (Hypoxico OHG, Traunstein, Germany) was set 12.6% mimicking an altitude of 4.500 meters. The study was drafted to stay for 12 h in the chamber. Measurements with all parameters were taken at 6 and at 12 h of continuous hypoxia.

Inside the hypoxia chamber air temperature was kept on a constant level of 20°C (72°F) to eliminate effects on microcirculation.

### Data collection

Hemodynamic parameters were collected with the subjects in sitting position. Pulse-oxymetric oxygen saturation measurements were performed with a fingertip pulse-oxymeter (Pulsox-3i Minolta, Osaka, Japan) calculating the mean value over a period of 5 min. An arterial blood gas analysis and arterial lactate measurement were performed on every participant at each time point. Calculating relative microvascular conductivity provides additional information (Cracowski et al., 2006; Pajk et al., 2017). Cutaneous vascular conductance was determined using the following formula: [BL/MAP].

The degree of acute mountain sickness was evaluated by using the LLS (Roach et al., 1993): Briefly, participants were asked to rate the symptom complexes headache, gastrointestinal symptoms, fatigue and/or weakness, and dizziness from 0 (not present) to 3 (severe). Summing all items up calculated a total score ranging from 0 to 12. Sleeping quality obviously was not included in this score as subjects did not sleep during measurements.

#### Microcirculatory measurements

Skin microvascular blood flow and reactive hyperemia response after arterial occlusion in the patient forearm were assessed by laser Doppler velocimetry (Periflux 4001, Perimed, Järfälla, Sweden). Reactive hyperemia is indicative of the autoregulatory capacity of the microcirculation responding to a reduction in oxygen delivery. Laser Doppler measurements are based on the principle that light, scattered by moving red blood cells, experiences a frequency shift proportional to the velocity of red blood cells. The Periflux 4001 uses laser light with a wavelength of 770–790 nm. A fiberoptic guide-wire (PF407, Perimed) conducts laser light to the tissue and carries back-scattered light back to a photodetector. Calibration of the laser Doppler flowmeter device was performed by using the manufacturer's original calibration kit (Perimed; Järfella; Sweden). Adjusting to zero was conducted on the surface of a white compact synthetic material (*PU* = 0). The second value of the calibration curve (*PU* = 250) was derived by measurement in a motility standard fluid provided by the manufacturer (Perimed; Järfella; Sweden). During the examination period, the electrode was placed on the volar aspect of the forearm and was held in place by adhesion force, generated by a surrounding thin transparent silicon rubber patch, approximately 2 cm in diameter using a self-adhesive ring. Skin microvascular blood flow was recorded in relative perfusion units (PU). Forearm ischemia was produced with a sphygmomanometer cuff wrapped around the arm over the brachial artery, and inflated to 200–220 mmHg for 3 min.

Pre-occlusive baseline PU (BL) was recorded for 3 min. During reperfusion, post-ischemic peak PU (Max) and the reperfusion PU (Rep) were measured. After measurements, the difference between MAX and BL as well as the difference between Rep and BL were calculated. To determine the magnitude of reactive hyperemia, the following formula was used: [100^*^(Rep-BL)/BL %]. Measurements were ended, when for a period of at least 5 min stable *PU*-values were recorded after reperfusion.

### Statistical analysis

This investigation was considered to be a pilot study and was conducted as an amendment to another study (Burtscher et al., 2014; Niedermeier et al., 2017a,b). Since there were no data available on how or if hypoxia affects skin microcirculation during a 12-h exposure, no power-analyzed sample size estimation was performed.

The normality assumption was tested using the Shapiro–Wilk test.

Continuous demographic and clinical data at baseline were compared by paired Student's *t*-test (Gaussian distribution). LLS is a categorical variable with 4 different values (0, 1, 2, 3) and was compared using Fisher's exact test. Significance was assumed at *p* < 0.05. All results are given as mean values plus/minus standard deviation, except for LLS reported as medians and interquartile ranges.

Panel data for BL and delta were analyzed using linear mixed effects models.

## Results

One subject left the hypoxic chamber before time point 360 min and nine additional subjects before time point 720 min. In the acclimatization group, the LLS as a marker for high altitude sickness increased at baseline from median 0 (interquartile range 0.5) to 2 (3) and 1 (3) at time points 360 and 720 min, respectively. In controls, the LLS increased from 0 (1) to 2 (2.75) and 1 (1), respectively (*p* < 0.001).

Table 1 shows demographic, hemodynamic parameters and blood gas analysis measurements at base line. No statistical significant differences except of systolic blood pressure (116 ± 12 vs. 128 ± 14 mmHg; *p* = 0.006) could be detected between groups.

**Table 1:** Demographic data at base line before entering the hypoxic chamber.

**Groups**	**Acclimatization (*n* = 23)**	**Controls (*n* = 23)**
**DEMOGRAPHIC DATA**		
Age	26.9 ± 6.2	26.1 ± 3.5
Height (cm)	172.7 ± 7.2	175.9 ± 9.6
Weight (kg)	67.6 ± 11.1	66.7 ± 8.5
BMI (kg/m^2^)	22.6 ± 2.8	21.5 ± 1.4
sex	12 male 11 female	14 male 9 female

*BMI reflects the body mass index, Values are means ± SD*.

### Results between groups with or without short-term acclimatization

No significant differences between subjects with short-term acclimatization in a hypoxic chamber and those without could be observed in the present investigation. A higher systolic blood pressure in the control group disappeared during the study period.

### Systemic hemodynamic variables and blood gas analysis

Initiation of a hypoxic and normobaric environment results in a significant decrease in mean arterial blood pressure concomitant with an increase in heart rate. The low partial pressure in oxygen in the chamber decreased arterial partial pressure of carbon dioxide (CO_2_) parallel with an increase in arterial pH as a consequence of a significant decrease in arterial PO_2_ values over the time. Arterial lactate levels did not change over time (Table 2).

**Table 2:** Lake louise score, hemodynamic parameters and blood gas analyses at base line before entering the hypoxic chamber.

**Groups**	**Acclimatization (*n* = 23)**	**Controls (*n* = 23)**
**LAKE LOUISE SCORE**		
LLS	0 (0.5)	0 (1)
**HEMODYNAMIC DATA**		
HF (1/min)	71.6 ± 9.2	70.6 ± 8.1
RRsys (mmHg)	116.2 ± 12.0	127.6 ± 14.0^*^
RRdia (mmHg)	68.6 ± 7.5	70.1 ± 10.5
RR MAP (mmHg)	84.4 ± 7.8	89.2 ± 9.9
**BLOODGAS ANALYSES**		
pH	7.4 ± 0.0	7.4 ± 0.0
PaCO_2_ (mmHg)	38.6 ± 3.2	39.6 ± 3.5
PaO_2_ (mmHg)	74.6 ± 5.5	76.7 ± 5.1
SO_2_ (%)	96.8 ± 0.9	96.7 ± 1.3
SaO_2_ (%)	97.4 ± 0.7	97.3 ± 1.1
Lactate (mg/dL)	1.5 ± 0.5	1.4 ± 0.4

*LLS, reflects Lake Louise score; HF, reflects heart frequency; RRsys, reflects systolic arterial blood pressure; RRdia, reflects diastolic arterial blood pressure; RR MAP, reflects mean arterial blood pressure; PaCO_2_, reflects arterial partial pressure of carbon dioxide; PaO_2_, reflects arterial partial pressure of oxygen; SO_2_, reflects transcutaneous saturation of oxygen; SaO_2_, reflects arterial saturation of oxygen*.

* *Denotes a p < 0.01 in intergroup comparison. Values are means ± SD, exept of LLS reported as median and interquartile range*.

### Microcirculatory blood flow and reactive hyperemia

**Figure 2:**
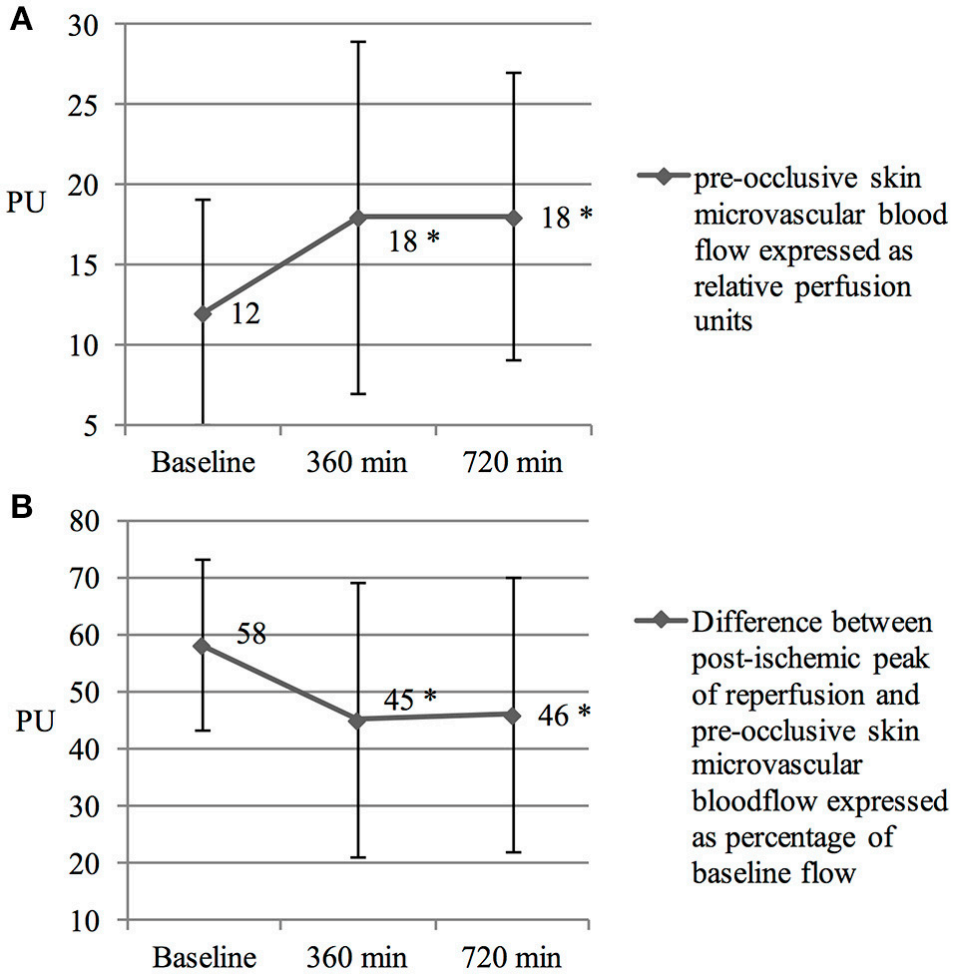
**(A)** Increased skin micro vascular blood flow. Skin microvascular blood flow was measured before hypoxic exposure, after 360 and 720 min in hypoxia, respectively. PU reflects perfusion units. ^*^Denotes a *p* < 0.01 in intragroup comparison. Values are means ± SD. **(B)** Diminished reactive hyperemia. Reactive hyperemia was measured before hypoxic exposure, after 360 and 720 min in hypoxia, respectively. PU reflects perfusion units. ^*^Denotes a *p* < 0.01 in intragroup comparison. Values are means ± SD.

Microcirculatory blood flow in the skin increased significantly over the time in all subjects (from 12 ± 7 to 18 ± 11, *p* < 0.001 and 18 ± 9 perfusion units, *p* < 0.001 at time point 360 and 720 min, respectively, Figure 2A).

Initiation of an ischemic stimulus resulted in a diminished reactive hyperemia response over time (from 58 ± 15% to 45 ± 24% and 46 ± 24% at time point 360 and 720 min, respectively; *p* < 0.001) in all subjects (Figure 2B).

## Discussion

In this study we evaluated the cutaneous microcirculatory answer to an ischemic stimulus in hypoxic, healthy subjects using this as a model for generalized microvascular responses. In a second leg of the investigation, we questioned whether intermittent hypoxia caused prolonged changes in cutaneous microcirculation or in other words circulatory acclimatization of the skin.

Our main findings were:

Cutaneous microcirculatory blood flow in hypoxia increases notably in this experiment, a known physiologic response (Haisjackl et al., 1990).
Looking at cutaneous vascular reactivity in hypoxia we saw that the response to an added ischemic stimulus is noticeably reduced.
Investigating for the first time—whether a 1-week preparation with hypoxia had an effect on blood flow—we could not find significant signs of acclimatization on the microvascular level.

### Acclimatization to hypoxia and microcirculation

Acclimatization mechanisms to hypoxia and high altitude are complex and include increased heart rate, hyperventilation, hypocapnia and a reduced renal conservation of bicarbonate. The oxygen-dissociation curve shifts to the left. Over time, the capillary density in tissues increases and so does the efficiency of oxidative processes in the mitochondria (Rowell, 1986).

No differences in microcirculatory parameters between subjects with and without hypoxic acclimatization could be found in the present study (Table 3).

**Table 3:** Systemic hemodynamic variables and blood gas analyses during hypoxia.

**Timeline →**	**Baseline before hypoxia**	**360 min in hypoxia**	**720 min in hypoxia**
**HF (1/min)**			
acclimatization	71.6 ± 9.2	81.5 ± 11.1^*^	77.6 ± 11.4
control	70.6 ± 8.1	81.4 ± 11.6^*^	80.5 ± 10.4^*^
**RRsys (mmHg)**			
acclimatization	116.2 ± 12.0	110.8 ± 10.7	119.0 ± 9.9
control	127.6 ± 14^†^	113.5 ± 11.1^*^	122.6 ± 9.8
**RRdia (mmHg)**			
acclimatization	68.6 ± 7.5	64.6 ± 6.3	76.7 ± 12.8^*^
control	70.1 ± 10.5	63.9 ± 6.9^*^	73.2 ± 4.5
**RR MAP (mmHg)**			
acclimatization	84.4 ± 7.8	80.0 ± 6.8	90.8 ± 10.6^*^
control	89.2 ± 9.9	80.4 ± 7.5^*^	89.7 ± 4.3
**pH**			
acclimatization	7.4 ± 0.0	7.5 ± 0.0^*^	7.5 ± 0.0^*^
control	7.4 ± 0.0	7.5 ± 0.0^*^	7.5 ± 0.0^*^
**PaCO**_2_ **(mmHg)**			
acclimatization	38.6 ± 3.2	32.0 ± 3.3^*^	31.4 ± 3.6^*^
control	39.6 ± 3.5	33.5 ± 3.1^*^	32.0 ± 3.2^*^
**PaO**_2_ **(mmHg)**			
acclimatization	74.6 ± 5.5	39.7 ± 4.0^*^	40.7 ± 3.7^*^
control	76.7 ± 5.1	38.5 ± 4.6^*^	39.4 ± 5.1^*^
**SO**_2_ **(%)**			
acclimatization	96.8 ± 0.9	79.4 ± 4.9^*^	81.7 ± 4.7^*^
control	96.7 ± 1.3	78.5 ± 6.0^*^	79.8 ± 7.0^*^
**SaO**_2_ **(%)**			
acclimatization	97.4 ± 0.7	83.7 ± 4.2^*^	85.5 ± 4.1^*^
control	97.3 ± 1.1	80.8 ± 5.0^*^	83.2 ± 6.3^*^
**LACTATE (mmol/L)**			
acclimatization	1.5 ± 0.5	1.7 ± 0.4	1.4 ± 0.4
control	1.4 ± 0.4	1.5 ± 0.3	1.2 ± 0.4

*HF, reflects heart frequency; RRsys, reflects systolic arterial blood pressure; RRdia, reflects diastolic arterial blood pressure; RR MAP, reflects mean arterial blood pressure; PaCO_2_, reflects arterial partial pressure of carbon dioxide; PaO_2_, reflects arterial partial pressure of oxygen; SO_2_, reflects transcutaneous saturation of oxygen; SaO_2_, reflects arterial saturation of oxygen*.

* *Denotes a p < 0.01 in intragroup comparison*,

† *Denotes a p < 0.05 in intergroup comparison. Values are means ± SD*.

One possible reason why we did not detect differences—if any existing—is that the applied hypoxic protocol (hypoxia at a FiO_2_ of 0.126 for 1 h over 7 days) was too short to cause changes in the skin microcirculation.

Similarly, the 7 days before testing may have been too long for a carry–over. A comparable short-term acclimatization protocol (at 3,650 m of altitude, ½ a mile lower than the 4,500 m in this investigation) failed to prevent mountain sickness in susceptible individuals at real altitude (Faulhaber et al., 2016). However, the hypoxic ventilatory response—seemingly the pivotal mechanism preventing acute mountain sickness—has been shown to last for up to 1 week, increasing SaO_2_ values up to 7% (Katayama et al., 2001, 2009). Furthermore, 7 days are usually needed for the journey from home to base camp of a climbing destination. It remains unclear if at all acclimatization in the cutaneous microcirculation is possible.

### Microcirculatory blood flow

In our study, normobaric hypoxia induced an increase in microcirculatory blood flow in the skin of healthy volunteers (Table 4). This response is in line with previous reports (Casey and Joyner, 2012; Paparde et al., 2014). In the setting of decreased oxygen delivery to the skin as in hypoxia the microvasculature reacts with a compensatory vasodilation. Here we observed an increased relative microvascular conductivity even with an increasing mean arterial pressure. As ambient pressure remains constant microcirculatory blood flow increases trying to equal oxygen supply with oxygen demand (Casey and Joyner, 2012).

**Table 4:** Microcirculatory blood flow and reactive hyperemia.

**Timeline →**	**Baseline before hypoxia**	**360 min in hypoxia**	**720 min in hypoxia**
**Rep (PU)**			
acclimatization	33.3 ± 15.5	68.5 ± 20.0	32.2 ± 17.7
control	28.0 ± 12.5	30.1 ± 11.5	32.2 ± 17.7
**Max (PU)**			
acclimatization	107.5 ± 42.7	104.2 ± 46.7	103.3 ± 43.0
control	89.2 ± 25.0	89.7 ± 36.1	86.3 ± 40.5
**Delta max (PU)**			
acclimatization	93.7 ± 41.8	86.0 ± 43.5	81.4 ± 40.4
control	78.3 ± 23.0	73.2 ± 37.3	70.2 ± 38.6
**CVC**			
acclimatization	0.16 ± 0.1	0.23 ± 0.1^†^	0.25 ± 0.12^†^
control	0.12 ± 0.1	0.21 ± 0.07^*^	0.18 ± 0.05^†^

*Rep reflects post-ischemic reperfusion of skin microcirculation expressed as relative perfusion units, Max reflects post-ischemic peak during reperfusion of skin microcirculation, Delta max reflects difference between post-ischemic peak of reperfusion and pre-occlusive skin microvascular bloodflow expressed as relative perfusion units. CVC reflects cutaneous vascular conductance*.

* *Denotes a p < 0.01 in intragroup comparison*.

† *Denotes a p < 0.05 in intergroup comparison. Values are means ± SD*.

The results of our laboratory study results are not in line with a study investigating four mountaineers at an altitude of 6,400 m (Martin et al., 2009). In this observational investigation microcirculatory blood flow was measured semi-quantitatively on the sublingual mucosa under the tongue. The main finding in this field observation was a more pronounced heterogeneous blood flow pattern in the capillaries. Heterogeneity was not assessed in our study; also we looked at skin rather than at a mucous membrane.

Moreover, Paparde and coworkers showed an increase of forearm skin blood flow during very short (20 min) bouts of systemic hypoxia with is in line with our results (Paparde et al., 2015). However, in this small study (eleven subjects), regional blood flow and vascular conductance remained unchanged during hypoxia (Paparde et al., 2015).

### Cutaneous adaption to hypoxia: a central or a local response?

During longer lasting hypoxic conditions the increase in blood flow parallel to a decrease in peripheral vascular resistance mirrors a regional compensation mechanism and is not influenced by a systemic adrenergic blockade (Richardson et al., 1967). The autonomic nervous system does not seem to play a major role in the adaptive mechanism of increasing microcirculatory blood flow. Investigations indicate a more local and regional control of blood flow regulation during decreased oxygen delivery. Seemingly, the endothelial cell lining of blood vessels and the smooth muscle cells independently mediate the microvascular responses to hypoxia (Kourembanas et al., 1998). Furthermore, during pronounced reductions of perfusion pressure vasomotor autoregulation in the forearm serves to maintain regional blood flow (Durand et al., 2004). Interestingly, there seem to be differences between races. For instance, the “Extreme Everest 2”-research group found a higher sublingual microcirculatory blood flow and an increased capillary density at high altitude in Sherpas as compared to lowlanders (Gilbert-Kawai et al., 2017).

### Reactive cutaneous hyperemia in hypoxia

Following complete occlusion a subsequent period of hyperemia occurs. In the present study the magnitude of this phenomenon termed reactive hyperemia was diminished during normobaric hypoxia in all persons (Figure 1). Reactive hyperemia is a marker of microvascular response to tissue hypoxia involving capillaries, arterioles, and small arteries. The increase in regional blood flow after vascular occlusion is directly related to the severity and duration of ischemia (Sparks and Belloni, 1978). Both the peak and the duration of the hyperemia depend on the duration of the occlusion, with the duration of the reactive hyperemia being especially pronounced after longer occlusions (Johnson et al., 1976). This relationship has led to the concept of “flow or oxygen-debt repayment,” where repayment signifies the area under the hyperemia curve (Coffman and Gregg, 1960; Olsson and Gregg, 1965; Johnson et al., 1976; Lombard and Duling, 1981).

This decreased responsiveness to endothelium dependent relaxation during a hypoxic state may be explained by an impaired vasodilatory reserve of the microvasculature in response to an ischemic stimulus (Sparks and Belloni, 1978; Knotzer et al., 2006). This phenomenon was only reported in clinical studies in patients with severe impairment of the physiological state like sepsis and multiple organ dysfunction syndrome (Haisjackl et al., 1990; Knotzer et al., 2006, 2007; Knotzer and Hasibeder, 2007, 2008). Why hypoxia and severe disease result in similar changes of microvascular reactivity is peculiar and deserves further investigations.

## Limitations

The present study was an amendment of a study investigating the effects of a simulation of high-altitude training and so no sample size calculation was performed. Nevertheless, microcirculatory parameters were calculated blinded (i.e., no knowledge of group membership).

A further limitation of this study is the fact that microcirculatory regulation differs between organs and even in different regions in one organ (Granger et al., 1983; Burtscher et al., 2014). This was not taken into account as well as possible interindividual differences of laser Doppler measurements. Different types of receptors in the vessels, receptor density and regional sympathomimetic mechanisms influences together with local metabolism the blood flow in tissues. This leads to a heterogeneous distribution of systemic blood flow in different organs. In the present study, we looked exclusively at the skin of the forearm.

As we sought to design a pilot study with measurements easily repeatable in the field (i.e., at high altitude), we did not perform near infrared spectroscopy. Data gathered from surface clark type electrodes from the human skin have to be interpreted with caution, as the penetration depth through the skin is limited (Knotzer and Hasibeder, 2007).

The 4 weeks-period between first familiarization to hypoxia and the experimental test run was chosen on a judgment based on our own (unpublished) data. However, further studies are needed to clarify this issue.

## Conclusion

The microcirculatory blood flow in the skin was increased during hypoxia probably due to an improvement in systemic cardiac output. The increased blood flow in the microcirculation was an answer to a decrease in chemically bound and physically solved oxygen in the blood.

Interestingly, the auto-regulative capacity of the microcirculation in the skin after an ischemic stimulus was considerably reduced under hypoxic conditions. The mechanism is unknown.

We could not find differences in microcirculatory blood flow and auto-regulative capacity of the skin microcirculation on forearm between subjects with and without hypoxic acclimatization. With this protocol, the skin does not acclimatize to hypoxia.

## Author contributions

BT, AK, HK, MB, K-HS, and WP conceived the ideas and designed this study. BT, AK, MB, IS, and MP collected the data. BT, AK, and MB analyzed and interpreted the data, drafted the manuscript, preparing the figures and tables. K-HS and HK revised the article critically. All authors finally approved the manuscript.

### Conflict of interest statement

The authors declare that the research was conducted in the absence of any commercial or financial relationships that could be construed as a potential conflict of interest.
